# General Remarks on Alterative Medicines

**Published:** 1851-02

**Authors:** W. S. Chipley

**Affiliations:** Lexington, Ky.


### WESTERN JOURNAL

OF

### MEDICINE AND SURGERY.

FEBRUARY, 1851.

---

Art. I.—*General Remarks on Alterative Medicines.* By W. S. Chipley, M. D., of Lexington, Ky.

Every candid practical observer, to whom the complex properties of medicinal substances are familiar, must be prepared to admit the futility of all attempts at a perfect and satisfactory classification of the Materia Medica. There is scarcely a medicinal agent which may not justly claim rank in more than one class, and there are not a few productive of such contradictory results as to render it extremely doubtful which is the most prominent characteristic. As has been remarked, medicines are often two-edged, cutting both ways. It becomes, then, the duty of the intelligent practitioner to seek to utilize the useful property, and to neutralize that which existing circumstances may render hurtful.

Notwithstanding this diversity of action, so characteristic of most medicinal agents, I believe that classification within certain limits may be extremely useful, especially in facilitating an acquisition of a knowledge of the Materia Medica, and in placing that knowledge more immediately at command in case of need.

Some of the articles comprised in the class, known as Alteratives, on which I design at present to make some general remarks, are peculiarly diversified in their effects; yet I think there will be no difficulty in making myself intelligible when I shall have clearly defined the property that affiliates these articles in one class under the denomination of Alteratives.

There are some medicines which seem to affect principally or uniquely the nervous system—evanescent in their effects, a few hours or days at most suffice to efface all trace of their action—no permanent modification remains to attest the power of the agent and the system resumes its wonted condition as if no disturbance had occurred. These agents may be powerful in the limited sphere in which they act, but there is no response in the general economy nor any permanency in the effects produced. There is a striking contrast between this class of remedial agents and that which I propose to consider. Less conspicuous primarily, alteratives are more durable in their effects. They happily fulfil indications that medicines of a fugacious character could not reach. They affect the organic elements of both the fluids and solids in a manner calculated to endure long after the primary impression of the medicine has passed away. This class of remedies formerly attracted much more attention, when acrimony was the most common reputed cause of disease, and alteratives then embraced many more articles than are now generally conceded to belong to the class. Periera still views this class as including "nearly the whole of the articles comprising our Materia Medica." I desire, however, to be understood as useing the term in a much more restricted sense, yet in a sense which will embrace a great variety of important articles. Definition, at all times difficult, is peculiarly so in this instance, as it is almost impossible to convey, in few words, the meaning of the term alterative without a feeling of being too contracted or too comprehensive. I will, however, venture to define the term as embracing those medicines that insensibly produce a profound impression on the organic elements, by which a salutary change is effected either in the solids, or fluids or both, calculated to neutralize certain morbid actions of the system, and thereby to restore health.

It was formerly supposed that these medicines acted chiefly by exciting and supporting the evacuation from the skin; but this opinion is now generally abandoned and will not therefore be considered.

At this day four opinions seem to divide intelligent members of the profession:

1. That alteratives have the property of altering, in some inexplicable manner, the nature or quality of vital action.

2. That their salutary influence is the result of a change effected in the composition of the organic tissues.

3. They are believed to expend their whole force on the fluids, altering their composition, consistency, etc., sometimes rendering them less adapted to interstitial nutrition, and less capable of furnishing the elements of acute or chronic inflammation.

4. That alteratives make a primary impression directly on the nervous system, affecting the general economy, and controlling morbid action only secondarily.

Each of these propositions expresses a truth, but nei- of them is exclusively correct. There is, in fact, no uniformity in the mode of action of different alteratives—the solidist who would limit the action of this class of medicines to the organic tissues, is not less grossly in error than the humoralist who would maintain that all alteratives exert an exclusive influence over the fluids. The truth of each of the above propositions will hereafter be illustrated by reference to the mode of action of particular articles belonging to this class.

Alteratives are indicated chiefly in the management of chronic disorders, but are sometimes useful in acute maladies of a grave character. In mild disorders, only slightly modifying the general economy, limited in extent, and affecting organs not absolutely essential to life, it will be readily perceived that our means may safely correspond with the unimportant character of the disease, and simple remedies suffice to effect a cure. But if graver morbid action invades one or more important organs, or speedy dissolution is threatened in consequence of the extent of diseased action affecting profoundly the whole economy, more energetic and powerful means are demanded, and duty enjoins a resort to those remedial agents that are known to exert not only an active, but durable influence.

Blood-letting has, by some authors, been placed at the head of alteratives, but this agent is with much more propriety classed with antiphlogistics. True, it does exert a powerful alterative influence, and the same may be said of almost every article of the Materia Medica; but it is not an alterative in the sense in which I desire to use that term. It is too prompt in its action—suddenly despoiling the system of a portion of the vital fluid, altering the composition of what remains, and thereby depriving the solid tissues of the elements of life on which their reparation depends. But when we repeat that alteratives, properly speaking are applicable, almost exclusively, to the treatment of chronic diseases, it will be at once perceived that blood-letting is wholly foreign to this important class of remedies, save as an adjuvant. For it should be remembered, that blood-letting, and many other remedial agents, may be resorted to as useful adjuvants in the course of an alterative treatment.

When alteratives are demanded in the management of acute disorders, particular regard must be had to the mode of action of the different articles which compose the class. In such cases the desideratum is usually either a modification of the crasis of the blood or a prompt alteration of morbid action. Certain alteratives, as iodine, arsenic, etc., always produce a certain degree of irritation, ever so much more intense as we seek a prompt and decided effect; there are others, however, that nettly fulfil the indication and will rarely disappoint the judicious practitioner. Saline remedies are alteratives in virtue of their property of attenuating the blood, while mercurials exert as decided an influence by changing the action of the extreme vessels. In puerperal peritonitis so decided is the influence of mercurials that many physicians regard them as the sheet-anchor of hope and safety. That the beneficial results in this case are fairly attributable to the alterative properties of the medicine I have no doubt, and I am confirmed in this opinion by the fact that the benefit is always more decided when the cathartic influence of the remedy is restrained by combination with opium. This is an instance in which the alterative acts by effecting a change in the nature and quality of vital action.

Gendrin has demonstrated the safety and utility of large doses of nitrate of potash in febrile articular rheumatism. In this case there is an alterative action as effectual as, but widely different from, that produced by mercury. The primary effect is a modification of the circulating fluids—an attenuation of the blood which enables the engorged and oppressed vessels to relieve themselves and recover their normal action.

But alteratives are comparatively little used in acute affections. They rise in importance in proportion as morbid action has been protracted and disease has struck deep its roots in the constitution, spreading from organ to organ, and giving rise to a series of consecutive accidents that points clearly to a lingering and painful existence, or to a speedy dissolution. Those medicines that produce only a temporary modification of the organism, however powerful in their primary operation, become useless, or only serve to flatter and deceive.

Most mineral waters probably owe their efficacy to the alteration they effect in the circulating fluids. Mr. E. A. Jennings says:

"Though great allowance must be made for the effects of change of air, relaxation from business, change of habits, and other accidental circumstances, yet all candid observers must acknowledge that, when due allowance has been made for all these circumstances, a large residue of cases remain where the most surprising good effects have been produced by a course of mineral waters. From the effects generally produced by them on the kidneys, it is evident that a large portion of the water is absorbed; and it is probable that their good effect is to be attributed as much to the alteration they produce in the chemical character of the blood, by entering into its composition, as to the increase they occasion of the secretions of the bowels."

Our remedies may be directed against causes or effects. Chronic maladies are often encountered when it is wholly impossible to trace the existing lesions to their source—the cause has disappeared, the effects only remain. Of course in this case no regard can be had to that of which we know nothing, and such agents must be selected as are best calculated to obviate the effects and restore healthy action. When the cause is appreciable and still exists, exerting its baneful influence, the first indication is to neutralize or remove it. The recuperative force of the living economy is often sufficiently active to restore health after the removal or neutralization of the cause, hence a thorough and minute analysis of the entire history of the case, with a view to discover the source of evil, cannot be insisted upon too strenuously. It must not be supposed that every chronic disease calls for the administration of alterative medicines; the cause of such maladies is often detected in vicious habits, an abandonment of which is a condition, without which a cure cannot possibly be effected, and a compliance with this condition will not unfrequently supersede the necessity for medicines. In such cases the exhibition of alteratives is useless and often positively injurious.

The importance of a careful scrutiny of each individual case will appear more clearly, when it is recollected that an alterative productive of the happiest effects, when directed to neutralize a cause, may prove highly injurious if given to obviate consecutive accidents. No remedy can be more certain or active in primary syphilis than mercury, yet it is scarcely possible to conceive of more lamentable results than flow from the use of the same remedy in the secondary forms of that loathsome scourge of vice. The preparations of Iodine exert little if any influence on scrofula in the primary stages of that terrible disease, but who has not been gratified with the wonderful operation of the same remedies when given to alleviate those grave organic alterations, effects of the scrofulous vice, that so often disfigure beauty, and render life a burden.

It has been said that in the mode of action of alteratives on constitutional vices, and on virus, there is something quite specific; there is nothing evident intermediate between the cause and effect; the effect of the medicine on the economy in health will not enable us to anticipate its anti-syphilitic or anti-scrofulous curative action. At the same time it is believed that when they are considered independent of their specific action, in relation to ordinary chronic disorders, their mechanism may, to a certain extent, be comprehended.

Now, I am of opinion that the mechanism of the so called specific action of mercury in the cure of syphilis, may be as rationally explained as the mechanism of this and other remedies in the cure of common chronic diseases.

It is said that alkalies are serviceable in engorgements of the liver, and their action is thus explained:—The function of this organ consists in the elaboration of the immense amount of blood that it is constantly receiving; when diseased, soda is administered by which the blood is rendered evidently alkaline, less coagulable, and thinner; thus facilitating the circulation in the hepatic vessels, and thereby placing the organ in the best possible condition to resume its normal offices.

This explanation may be correct, but it is not a whit more reasonable than that which explains the mechanism of mercury in the cure of syphilis, by attributing its salutary influence to the change it effects in the morbid action that generates the virus of this disease. This change consists in the substitution of a harmless action tending to health, for one which, unchanged, surely terminates in disorganization and death.

But some able writers have stoutly maintained the doctrine that many chronic diseases, as well as some acute disorders, are caused by accidental productions, similar to fungi, mosses, and mushrooms. Morbid seed germinate in the economy at the expense of the fluid, and the fruit is matured in the substance of the tissues or on the surface, constituting those local lesions which are troublesome to the sufferer, either mechanically or in consequence of the inflammation they produce, the reabsorption of the products into which they are resolved, or of which they provoke the secretion. This pathology is said to be easily demonstrated as applicable to most of the diseases of vegetables, and Trousseau declares that the day may come when these views will not appear altogether absurd in regard to man and animals.

Taking this as a starting point, he says, it is easy enough to explain how alteratives act in curing most chronic maladies, such as tetter, cancer, scrofula of which the cause is destroyed in the same manner as certain poisons destroy fungi, mosses, or mushrooms, which are developed on organic elements in decomposition or even on those not yet in decay.

The truth or falsity of this theory cannot be determined in the present state of medical science, but it undoubtedly throws open an extensive field for cultivation, where philosophical observers may hereafter win enduring fame for themselves and confer lasting benefit on their race.

I have already admitted that no rigorous classification of the Materia Medica can be made; antim. tart., for instance, is very properly classed with emetics, but it may act as a sudorific, expectorant, or cathartic. The same diversity of action characterizes alteratives; calomel is one af the most powerful of the class, but it may also act as a cathartic or sialogogue. This fact suggests some useful cautions. It is known to every practitioner of experience, that when an alterative acts with violence on the primæ viæ, or produces any considerable evacuation, its energies are uselessly expended, and the object of its exhibition defeated. Hence the importance of observing effects closely, so as to be prepared to limit the medicine to its alterative influence, by such judicious combinations or modification of doses or preparation, as each individual case may demand.

Again, it is not a matter of indifference what article shall be selected for a particular case, for the action of these medicines is far from being identical. Some of them make a profound impression on the constitution, enfeeble the general system, and may not be wholly neutralized for months; while others, acting apparently with equal power, are more readily assimilated or eliminated, a few days sufficing for the disappearance of any depressing influence they may exert. Hence the propriety, especially in acute disorders, of selecting the former in the treatment of vigorous persons in whom reaction is intense and inflammatory action continuous; while the latter should be reserved for those patients whose constitution gives reason to apprehend collapse after the abatement of the disease.

Peculiarity of constitution may interdict the use of alteratives, that are used with the greatest advantage in the treatment of the same disease as it occurs with a vast majority of persons. Iodine and its preparations are applicable to the treatment of a great variety of chronic maladies, yet I have met with patients who could take only certain preparations of this medicine, and others who could not, without positive and serious injury, take it in any form, although general experience had pointed it out as the most efficient remedy for the disease with which the sufferers were afflicted.

As a general rule alteratives are administered only in small doses, and require to be continued a considerable length of time, in order to affect favorably the organism and to restore healty action to the solids or normal properties to the fluids. Small doses are in most cases absolutely necessary to secure the alterative effect of the medicine, with which some other of its medicinal properties may be wholly inconsistent. Mercury in small doses is a powerful alterative—in large ones cathartic, and if the latter effect is obtained, the former is lost. Homœopathic remedies, when they possess any activity whatever, must owe all their efficiency to their alterative properties. But that absurd system owes far more to the recuperative power of nature, under a rigid regulation of diet, clothing, exercise, etc., than to any active property of the infinitesimal doses with which its supporters may be pleased to amuse the imagination of their victims.

The same assiduous attention, to what our fathers termed the non-naturals, is imperatively demanded in every case that calls for the exhibition of an alterative. A strict attention to diet, rest, exercise, excretions and affections of the mind constitutes an essential feature in every rational plan of alterative treatment, without which, in grave cases, no reasonable hope of cure can be entertained. I have never known a practitioner, celebrated for unusual success in the management of chronic disorders, who was not remarkable for the extreme rigor with which he enforced a proper regimen.

I do not pretend to claim any novelty for the views expressed in this paper, and would be very far from insisting on the theoretical opinions expressed. It is consoling to reflect that whether these theoretical opinions be true or false, the correctness of the practical deductions is unaffected; for these deductions are made from experience, and do not follow, but precede theory. Whether the mode of action of mercury consists in altering or suspending morbid action, or that of alkalies in attenuating the blood, or that of prussic acid in a direct impression on the nervous system, experience has demonstrated that these medicines are peculiarly appropriate to certain disorders, and that they cannot be successfully or safely employed indiscriminately. Each individual case of disease must be thoroughly studied in all its phases; the constitution, hereditary taint, habits, condition, profession, idiosyncracy, etc., of the patient must be studiously canvassed before the intelligent practitioner will be prepared to select his remedy. Having thus made the selection, another series of observation is imperatively demanded. Every effect must be carefully noted, and the remedy be continued, modified or abandoned, as each case may require.

Without this system of laborious and untiring observation and reflection, little may be hoped for in the management of grave chronic affections. Accident, may indeed, sometimes supply these high and ennobling qualities of the scientific physician; but there can be neither the same safety or security for the patient, or satisfaction to the practitioner.

